# B7-H3 as a promising target for cytotoxicity T cell in human cancer therapy

**DOI:** 10.18632/oncotarget.8784

**Published:** 2016-04-18

**Authors:** Juan Ma, Pan Ma, Chenghai Zhao, Xin Xue, Huamin Han, Changzhen Liu, Hua Tao, Weigang Xiu, Jia Cai, Man Zhang

**Affiliations:** ^1^ Department of Clinical Laboratory Medicine, Beijing Shijitan Hospital, Capital Medical University, Key Laboratory of Urinary Cellular Molecular Diagnostics, Beijing, China; ^2^ Key Laboratory of Protein and Peptide Pharmaceuticals, Institute of Biophysics, Chinese Academy of Sciences, Beijing, China; ^3^ Department of Pathophysiology, College of Basic Medical Science, China Medical University, Shenyang, China; ^4^ Department of Immunology, China Basic Medical Theory of Chinese Medicine, Academy of Chinese Medical Sciences, Beijing, China; ^5^ CAS Key Laboratory of Pathogenic Microbiology and Immunology, Institute of Microbiology, Chinese Academy of Sciences, Beijing, China; ^6^ Department of Molecular Biology, Experimental Research Center, China Academy of Chinese Medical Sciences, Beijing, China; ^7^ Chinese Medical Doctor Association of Lab Medicine, Beijing, China

**Keywords:** B7-H3, bispecific antibody, immunotherapy

## Abstract

Targeting B7-H3 over-expressed tumor cells with anti-B7-H3 monoclonal antibodies inhibits tumor growth. Here we demonstrated the expression of B7 family homologue 3 (B7-H3) in a wide range of human tumor cells and further investigated whether B7-H3 could be served as a target for T-cell mediated immunotherapy against human cancers. The specific cytotoxic activity of activated T cell (ATC) armed with a novel anti-CD3 x anti-B7-H3 bispecific antibody (B7-H3Bi-Ab) against tumor cell was evaluated in vitro and in vivo. In contrast with unarmed ATC, an increase in cytotoxic activity of B7-H3Bi-armed ATC against tumor cells was observed at effector/target (E/T) ratios of 5:1, 10:1, and 20:1. Moreover, B7-H3Bi-armed ATC secreted more IFN-γ, TNF-α and IL-2 than unarmed ATC. Infusion of B7-H3Bi-armed ATC inhibited tumor growth in severe combined immunodeficiency (SCID) xenograft models, along with a significant survival benefit. Therefore, treatment with novel B7-H3Bi-armed ATC will be a promising strategy for current cancer immunotherapy.

## INTRODUCTION

Following operation, chemotherapy, and radiotherapy, immunotherapy has been recognized as the fourth antitumor modality [1–3]. T cells recognize tumor cells through specific T-cell receptor (TCR), and serve as the promising effector cells for adoptive cell therapy. Over decades, researchers have made great efforts to take advantage of T-cell potency against tumor targets [4]. Recent successes in immune checkpoint blockade such as targeting CTLA-4 (cytotoxic T-lymphocyte antigen 4) and/or PD-1/PD-L (programmed cell death 1/PD-1 ligand) have fueled the treatment for cancers [5]. Another effective strategy is the administration of bispecific antibodies (BiAbs) that target TCR on one hand and the tumor-associated antigen (TAA) on the other [6].

Developed two decades ago, bispecific antibodies targeting different TAAs including EGFR, Her2, GD2, CD19, CD20, CD30, CEA, CA125, PSA, and EpCAM, have been tested in both experimental and clinical studies with encouraging results [6–9]. B7 family homologue 3 (B7-H3), a transmembrane protein with immunoglobulin-like structure, belongs to the B7 superfamily that activate or inhibit T-cell responses. The expression of B7-H3 protein maintains at lower levels in normal tissues whereas higher levels in many human malignancies, including neuroblastoma, melanoma, glioma, and lung, pancreatic, renal, colon, ovarian, breast, gastric, hepatocellular, colorectal, prostate, endometrial, and cervical cancer [10–13]. Researches have also demonstrated soluble B7-H3 (sB7-H3) in the serum of tumor patients [14, 15].

Despite conflicting effects of B7-H3 observed in some tumors, overwhelming studies revealed that B7-H3 exhibited inhibitory actions on host T cells in cancer patients [11, 13, 16, 17]. B7-H3-deficient mice developed experimental autoimmune encephalomyelitis several days earlier than their wild-type littermates [18]. The receptor for human B7-H3 on T cells is still unknown. Similar to CTLA-4, Tim-3, and PD-1, it is supposed to be a co-inhibitory receptor in humans. Consistently, its expression is usually associated with worse outcomes [11, 12, 17, 19, 20]. B7-H3 is considered as a TAA which regulates important cellular responses, including proliferation, apoptosis, adhesion, and metastasis [19–22]. Specific blockade of B7-H3 has been shown to inhibit tumor growth. MJ18, an anti-B7-H3 mAb, remarkably inhibited tumor growth in a pancreatic cancer model [11, 16]. Clinically, 8H9, another anti-B7-H3 mAb, was firstly applied in neuroectodermal tumors [11, 23]. Moreover, ^131^I-8H9 therapy, intrathecal injection 8H9 radiolabeled with ^131^I, has also shown efficacy in the treatment of metastatic neuroblastoma [24]. Recently, a phase I trial of anti-B7-H3 antibody (MGA271) is underway in the treatment of multiple refractory solid tumors that express B7-H3 [25, 26]. Collectively, these results indicate that B7-H3 represents a promising target for immune-based antitumor therapies.

In this study, clinically approved anti-CD3 antibody was chemically conjugated with anti-B7-H3 mAb antibody. The anti-CD3 x anti-B7-H3 bispecific antibody (B7-H3Bi-Ab) was then used to direct the activated T cell (ATC) to kill tumor targets. Armed with B7-H3Bi-Ab, ATC exhibited increased specific cytotoxicity and cytokine production, and suppressed B7-H3-positive cancer growth in SCID-Beige mice model.

## RESULTS

### B7-H3 overexpression in human cancer cells

**Figure 1:**
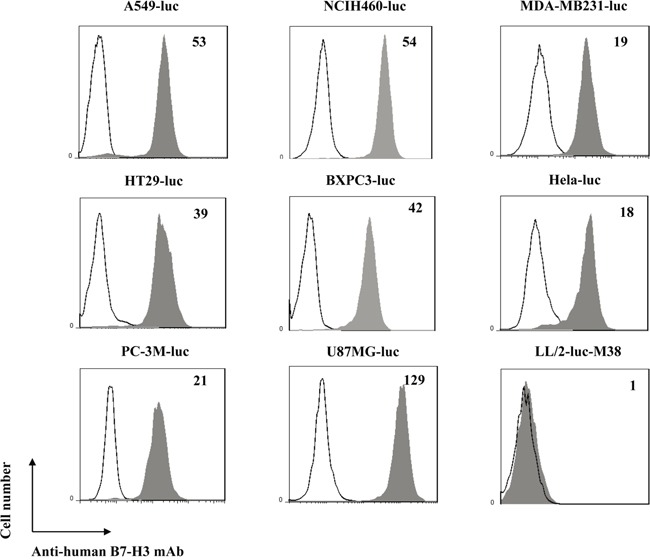
Expression of B7-H3 on different human tumor cells Shaded histogram represents cells stained with anti-B7-H3 mAb and un-shaded histogram represents cells stained with the control mouse IgG1. Mean Fluorescent Intensity (MFI) values obtained with anti-B7-H3 mAb staining divided by the control isotype staining are indicated in the upper right of the histogram.

B7-H3 expression in human tumor cells was assessed by FACS analysis including lung cancer (A549-luc and NCIH460-luc), breast cancer (MDA-MB231-luc), colorectal cancer (HT-29-luc), pancreatic cancer (BXPC3-luc), cervix cancer (Hela-luc), prostate cancer (PC-3M-luc) and glioblastoma (U87MG-luc). As shown in Figure 1, mean fluorescence intensity (MFI) values obtained with anti-human B7-H3 mAb staining divided by control antibody staining was indicated in the upper right of the histogram, and high B7-H3 expression was detected in all the human cancer cells. However, the anti-human B7-H3 mAb could not react with B7-H3 molecule on the surface of mouse lung cancer cell, LL/2-luc-M38, indicating the specificity of the anti-human B7-H3 mAb for human B7-H3 molecule.

### Preparation and characterization of B7-H3Bi-Ab and ATC

**Figure 2:**
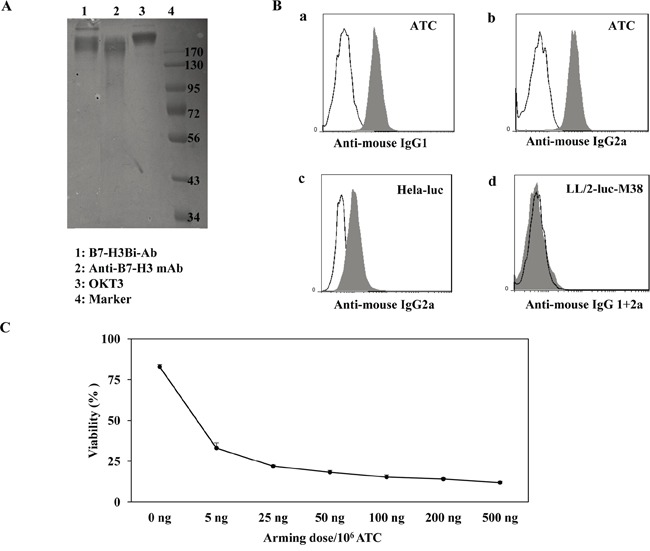
Generation of anti-CD3 × anti-B7-H3 bispecific antibody (B7-H3Bi-Ab) **A.** The heteroconjugated product of equimolar concentrations of B7-H3Bi-Ab was quantified by Coomassie blue staining of SDS-gel. **B.** Flow cytometry based binding assay for B7-H3Bi-Ab was tested. ATC was stained by B7-H3BiAb (shaded histogram), or a combination of OKT3 with anti-B7-H3 (the black line), then FITC-anti-mouse-IgG1 was added to detect the B7-H3 moiety of B7-H3Bi-Ab (a), or an FITC-anti-mouse IgG2a to detect the anti-CD3 moiety of the B7-H3Bi-Ab (b). (c) Hela-luc cells were incubated with B7-H3Bi-Ab (shaded histogram) or a combination of OKT3 and anti-B7-H3 (the black line), then B7-H3Bi-Ab binding was confirmed by FITC-anti-mouse IgG2a to detect the anti-CD3 moiety of the B7-H3Bi-Ab. (d) LL/2-luc-M38 cells were incubated with B7-H3Bi-Ab (shaded histogram) or a combination of OKT3 and anti-B7-H3 (the black line), then B7-H3Bi-Ab binding was analyzed by combined FITC-anti-mouse IgG2a with an anti-mouse-IgG1-FITC. **C.** Titer of B7-H3Bi-Ab armed ATC was measured. ATC was armed with B7-H3Bi-Ab ranging from 5 to 500 ng/10^6^ cells at E/T ratio of 10:1, and cytotoxic effects of B7-H3Bi-armed ATC on Hela-luc cells were tested in vitro. After 18 hour incubation with B7-H3Bi-armed ATC, the percentage of viability was calculated at each concentration.

Anti-human B7-H3 mAb was hetero-conjugated with OKT3 chemically named as B7-H3Bi-Ab. The hetero-conjugated product of equimolar concentrations of B7-H3Bi-Ab was quantified by Coomassie blue staining of SDS-gel as shown in Figure 2A. Densitometric quantitation of Lane 1 of the gel showed 73.5% monomer, 18.5% dimer, and 8% multimer fractions. The dual binding specificity of B7-H3Bi-Ab was tested (Figure 2B). ATC was stained by B7-H3Bi-Ab firstly, then an anti-mouse-IgG1-FITC was added to detect the B7-H3 moiety of B7-H3Bi-Ab. Only functional B7-H3Bi-Ab could bind to ATC by CD3-recognized OKT3 (isotpye: mouse IgG2a) and be detected through anti-B7-H3 mAb (isotpye: mouse IgG1) by anti-mouse IgG1 antibody. As shown in Figure 2B, positive stained cells were detected in 97% of the ATC population with a MFI of 10 (a). Moreover, the binding of B7-H3Bi-Ab to ATC was also evaluated by FITC-anti-mouse IgG2a (b). On the other hand, to evaluate the binding of B7-H3Bi-Ab to B7-H3^+^ cells, Hela-luc cells were incubated with B7-H3Bi-Ab, and the binding of B7-H3Bi-Ab to B7-H3^+^ cells was confirmed by FITC goat-anti-mouse IgG2a (c). In contrast, B7-H3Bi-Ab could not bind to CD3^−^B7-H3^−^LL/2-luc-M38 cell (d). Next, the amount of B7-H3Bi-Ab required to arm ATC was examined (Figure 2C). ATC was armed with B7-H3Bi-Ab ranging from 5 to 500 ng/10^6^ cells, and cytotoxic effects of B7-H3Bi-armed ATC on Hela-luc cells were tested in vitro. After 18 hour incubation with B7-H3Bi-armed ATC, the percentage of viability was almost 20% at the concentration of 50 ng B7-H3Bi-Ab/10^6^ cells at effector-to-target (E/T) ratio of 10:1, and the viability did not decrease significantly at the concentration of 500 ng B7-H3Bi-Ab/10^6^ cells.

### Cytotoxity effects of B7-H3Bi-armed ATC on different tumor cell lines

**Figure 3:**
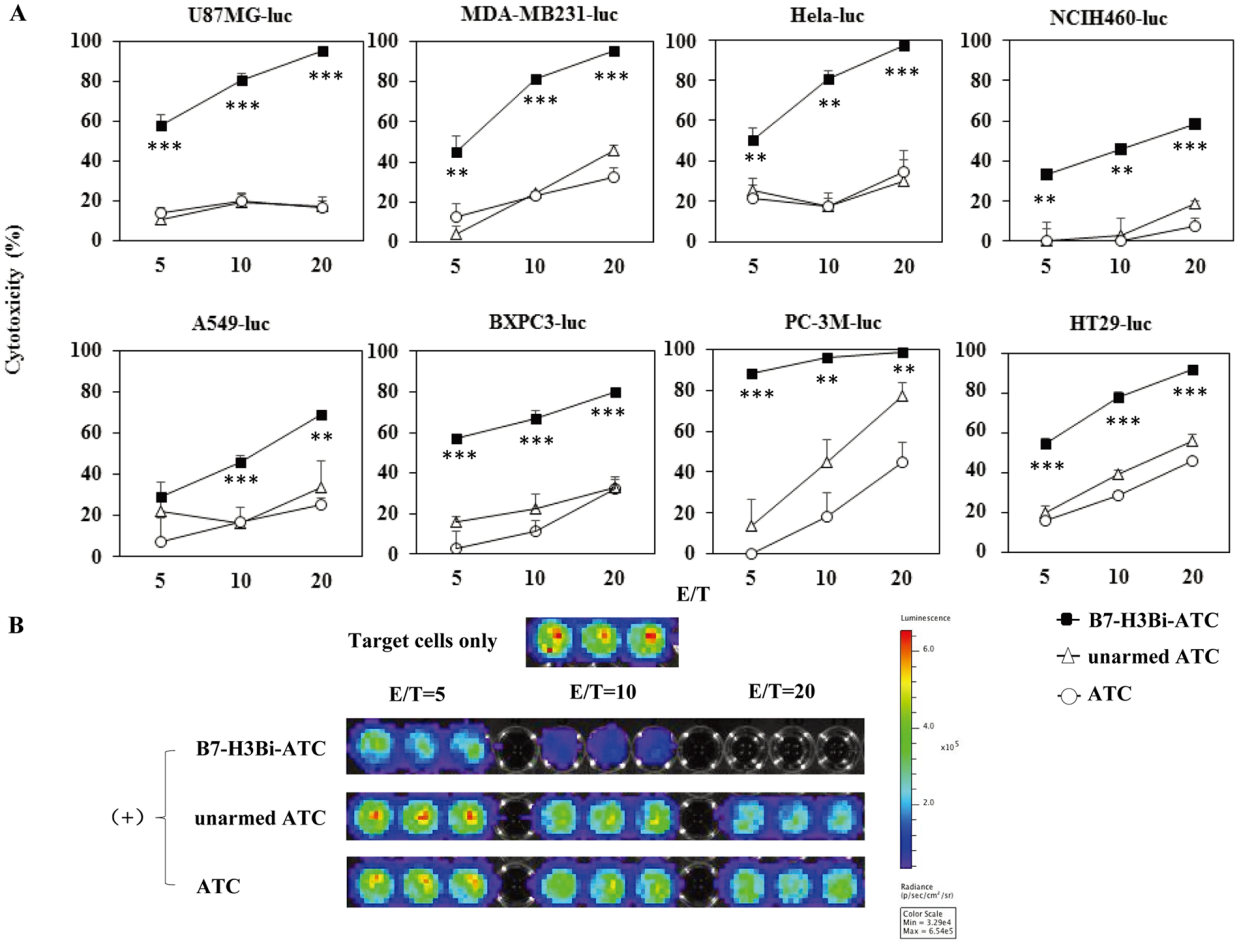
Cytotoxicity effects of B7-H3Bi-armed ATC against different tumor cells Target cells were incubated either with B7-H3Bi-armed ATC or a combination of OKT3 and anti-B7-H3 mAb with ATC (unarmed ATC), or ATC for 18 hours, and luciferase quantitative assays were performed to determine cytotoxicity against different target cells at indicated E/T ratio. **A.** Bioluminescence image signal in photons per second was converted into living cell number and the cytotoxicity assays were measured at the at different E/T ratio (5:1, 10:1, and 20:1). **B.** Bioluminescence image of MDA-MB231-luc cell was as a representative after incubation with B7-H3Bi-armed ATC or unarmed ATC, or ATC at the indicated E/T ratio. Shown is a representative experiment of at least three, and the data for (A) are mean ± SD of triplicate determination. **P < 0.01, ***P < 0.001, B7-H3Bi-armed ATC was compared with unarmed ATC or ATCunder similar conditions.

Since 50 ng and 500 ng/10^6^ cells showed a similar cytotoxicity, we chose 50 ng/10^6^ ATC as the concentration of B7-H3Bi-Ab for all subsequent experiments, and ATC mixed with both individual OKT3 and anti-B7-H3 mAb was used as unarmed ATC control. Cytotoxic effects of B7-H3Bi-armed ATC on different B7-H3^+^ tumor cells were tested in vitro, and the assays were performed at E/T ratios of 5:1, 10:1 and 20:1. After 18 hour incubation with B7-H3Bi-armed ATC, unarmed ATC, or ATC, as shown in the Figure 3A, the percentage of cytotoxicity with armed ATC was significantly greater than that with unarmed effectors at E/T ratio from 10:1 and 20:1 in U87MG-luc, MDA-MB231-luc, Hela-luc, NCIH460-luc, A549-luc, BXPC-3-luc, PC-3M-luc, and HT29-luc cells. The representative of bioluminescence image correlated with the number of living target cells was shown in Figure 3B. On the contrary, there was no difference in the bioluminescence imaging signal of the mouse lung cancer cell LL/2-luc-M38 after incubation with B7-H3Bi-armed ATC or unarmed ATC control (Supplementary Data 1).

### Cytokine production by B7-H3Bi-armed ATC

**Figure 4:**
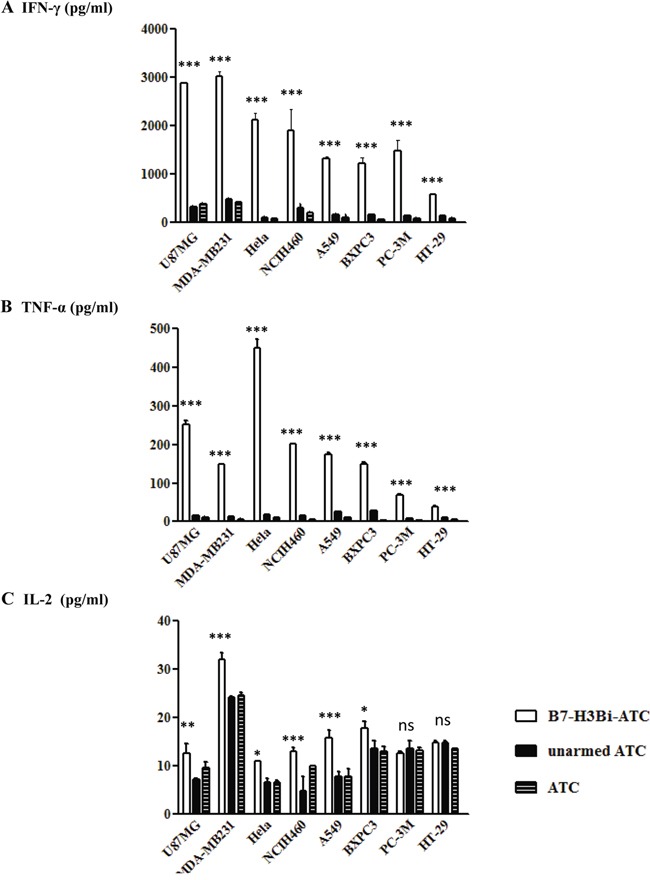
IFN-γ **A.**, TNF-α **B.**, and IL-2 **C.** secretion by B7-H3Bi-armed ATC against different tumor cells Supernatants of co-cultures at E/T of 10:1 were harvested at 18 hours and analyzed for cytokine production using specific ELISA Kit. The data are mean ± SD of triplicate determination. Shown is a representative experiment of at least three experiments. *P < 0.05, **P < 0.01, ***P < 0.001, B7-H3Bi-armed ATC was compared with unarmed ATC or ATC under similar conditions.

To analyze the levels of T cell-derives cytokines involved in cytotoxicity, cell supernatants were analyzed for IFN-γ, TNF-α and IL-2 production at E/T ratio of 10:1. As shown in Figure 4, a significant increase was observed in IFN-γ (Figure 4A), TNF-α (Figure 4B) and IL-2 (Figure 4C) secretion by B7-H3Bi-armed ATC over their unarmed ATC counterpart when ATC was co-cultured with U87MG-luc, MDA-MB231-luc, Hela-luc, NCIH460-luc, A549-luc, and BXPC-3 cells, respectively (*P* < 0.05). Given the possibility that the unarmed ATC also secreted considerable IL-2 when co-cultured with PC-3M-luc and HT-29-luc cells, no further increase was observed in IL-2 secretion when B7-H3Bi-armed ATC was co-cultured with them, although a significant increase was detected in IFN-γ and TNF-α production by B7-H3Bi-armed ATC over unarmed ATC counterpart. Interestingly, the unarmed ATC also showed substantial cytotoxicity when co-cultured with PC-3M-luc and HT-29-luc cells at E/T ratio of 10 and 20 (Figure 3).

### B7-H3Bi-armed ATC inhibited hela tumor growth in SCID-Beige mice

**Figure 5:**
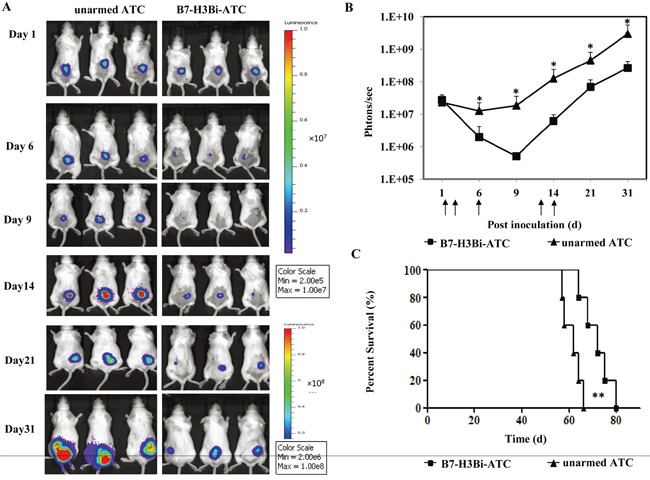
In vivo anti-tumor ability of B7-H3Bi-armed ATC in mouse subcutaneous cancer model SCID/Beige mice were inoculated subcutaneously with 1×10^6^ Hela-luc cells. On day 1, 2, 6, 12 and 14, tumor-bearing mice were locally treated with 2×10^7^ B7-H3Bi-armed ATC or unarmed ATC (each group contains 5 mice). Tumor growth was monitored using an in vivo imaging system **A.** Bioluminescence images of 3 representative mice of each group were shown on indicated day. **B.** Images were analyzed using Living Image software and tumor values represented as total flux measurements in photons/second, mean values of tumor growth curves were shown. **C.** Survival curves of mice engrafted with Hela-luc tumor cells receiving B7-H3Bi-armed ATC or unarmed ATC were shown. *P < 0.05, **P < 0.01, B7-H3Bi-armed ATC was compared with unarmed ATC under similar conditions.

To determine whether B7-H3Bi-armed ATC could suppress tumor growth in vivo, SCID-Beige mice were engrafted subcutaneously with Hela-luc cells. From the following day, mice were treated with B7-H3Bi-armed ATC or control unarmed ATC locally as indicated. The growth of tumor was monitored with bioluminescent imaging. In Figure 5A, three representative mice of each group were shown. Tumors grew consistently in mice receiving control unarmed ATC. On the contrary, mice receiving B7-H3Bi-armed ATC experienced a rapid tumor regression within 9 days of injection, and the tumor growth in this group was significantly delayed (Figure 5B). These results showed that B7-H3Bi-armed ATC can inhibit the tumor growth in vivo. Finally, a significant survival advantage was observed after the treatment with B7-H3Bi-armed ATC over that with control unarmed ATC (Figure 5C). Median survival time of the mice receiving the B7-H3Bi-armed ATC and unarmed ATC was 72 d and 62 d, respectively (*P* < 0.01).

### B7-H3Bi-armed ATC inhibited A549 tumor growth in SCID-Beige mice

**Figure 6:**
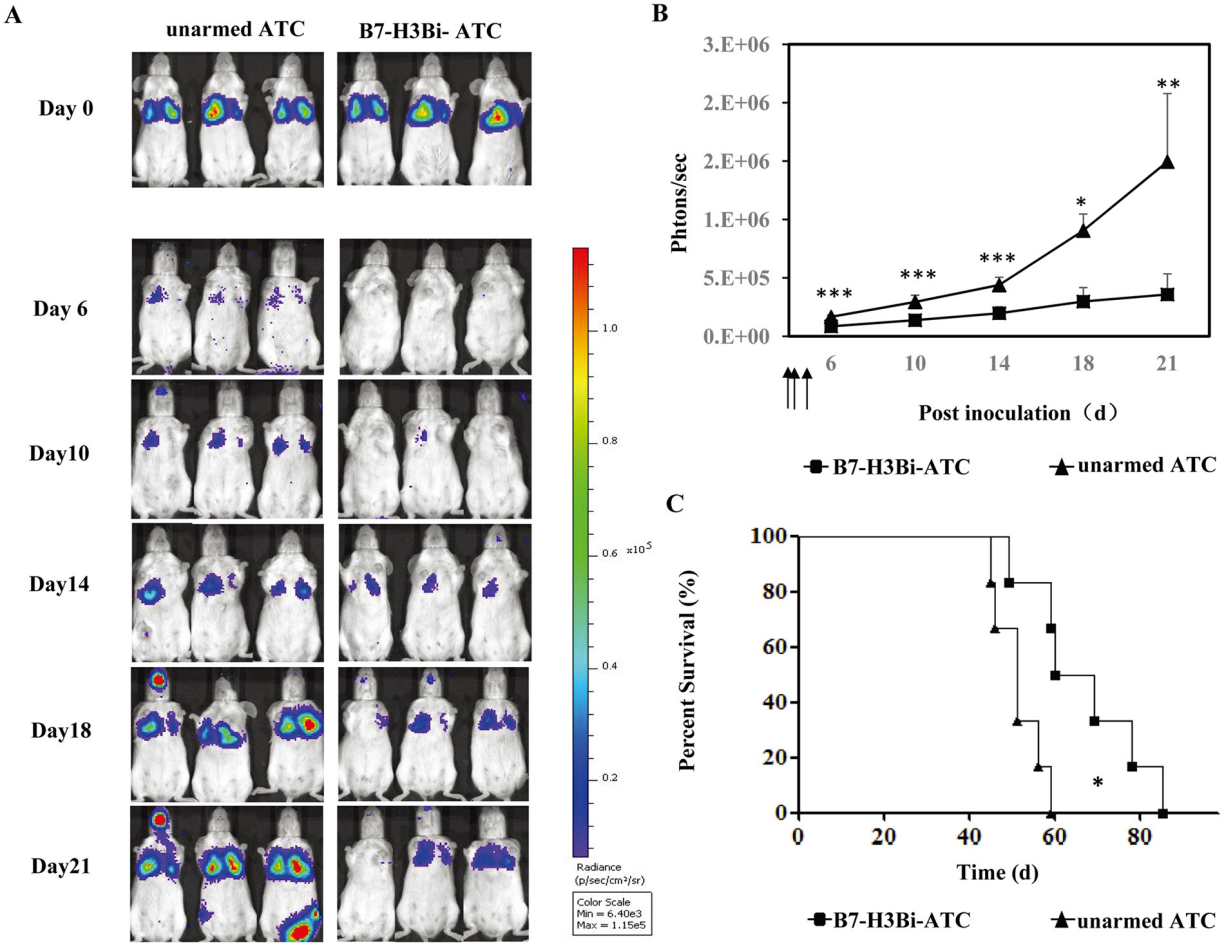
In vivo anti-tumor potency of B7-H3Bi-armed ATC in mouse lung cancer metastasis model SCID/Beige mice were inoculated intravenously with 2 ×10^6^ A549-luc cells. On day 0, day1 and day3, tumor-bearing mice were administrated i.v. with 2 ×10^7^ B7-H3Bi-armed ATC or unarmed ATC respectively (each group contains 5 mice). Tumor growth was monitored using an in vivo imaging system **A.** Bioluminescence images of 3 representative mice of each group were shown on indicated day. **B.** Images were analyzed using Living Image software and tumor values represented as total flux measurements in photons/second, mean values of tumor growth curves were shown. **C.** Survival curves of mice engrafted with A549-luc cells receiving B7-H3Bi-armed ATC or unarmed ATC were shown. *P< 0.05, **P < 0.01, ***P < 0.001, B7-H3Bi-armed ATC was compared with unarmed ATC under similar conditions.

To further determine whether B7-H3Bi-armed ATC could prevent metastatic tumor growth in vivo, SCID-Beige mice were engrafted intravenously with A549-luc cells. After inoculation, mice were divided into two groups randomly and treated with B7-H3Bi-armed ATC or control unarmed ATC intravenously on day 0, day 1 and day3. In Figure 6A, three representative mice of each group were shown. The strong light signal gathered in the lung showed the successful inoculation on day 0. Tumors grew consistently from day 6 in mice receiving control unarmed ATC. In contrast, mice receiving B7-H3Bi-armed ATC experienced remarkable tumor inhibition, and the tumor growth in this group was significantly delayed. The mean bioluminescence signal of each test group correlated with the number of living A549-luc cells was shown in Figure 6B. Finally, a significant survival advantage was observed after the treatment with B7-H3Bi-armed ATC over that with control unarmed ATC (Figure 6C). Median survival time of mice receiving the B7-H3Bi-armed ATC and control unarmed ATC was 67 d and 51 d, respectively (*P* < 0.05).

### Cytotoxity effects of B7-H3Bi-armed ATC on freshly isolated tumor cells from patients

**Figure 7:**
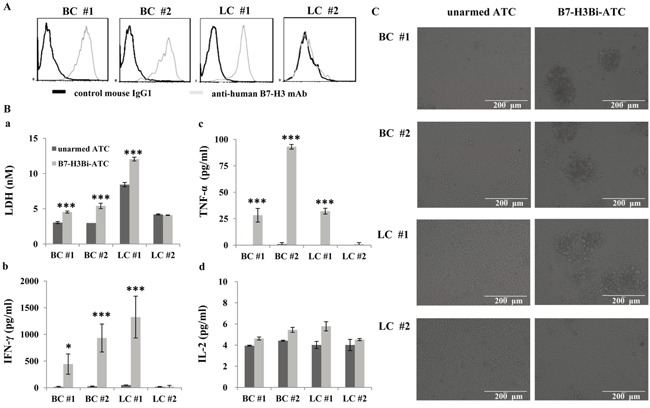
The cytotoxicity effects of B7-H3Bi-armed ATC against freshly isolated tumor cell from patients **A.** Expression of B7-H3 on human primary breast cancer (BC) and lung cancer (LC) cell culture. Gray histogram represents cells stained with anti-human B7-H3 mAb and black histogram represents cells stained with the isotope control mAb. **B.** (a) Lactate dehydrogenase (LDH) activity assay was performed by B7-H3Bi-armed ATC or unarmed ATC against freshly isolated tumor cell. Supernatants of co-cultures at E/T ratio of 10:1 were harvested at 18 hour and LDH activity assay was performed to determine cytotoxicity against freshly isolated tumor cell. (b) IFN-γ, (c) TNF-α and (d) IL-2 secretion by B7-H3Bi-armed ATC against freshly isolated tumor cell. Supernatants of co-cultures at E/T ratio of 10:1 were harvested at 18 hours and analyzed for cytokine production using specific ELISA Kit. **C.** Target tumor cells were incubated either with B7-H3Bi-armed ATC or unarmed ATC for 18 h at E/T ratio of 10:1, and real-time photograph was taken at x 200 magnification. The data of (B) are mean ± SD of triplicate determination. Shown is a representative experiment of at least three experiments. *P < 0.05, ***P < 0.001, B7-H3Bi-armed ATC was compared with unarmed ATC under similar conditions.

Finally, tumor cells derived from primary lung cancer and breast cancer patients were tested to evaluate whether they also expressed high levels of B7-H3 proteins. As shown in Figure 7A, B7-H3 positive stained cells were detected by FACS analysis in two breast cancer cell populations (BC #1 and BC #2) and one lung cancer cell population (LC #1), but not in the other lung cancer cell population (LC #2). Next, B7-H3Bi-armed ATC was tested for cytotoxicity on freshly isolated tumor cells. Lactate dehydrogenase (LDH) activity assays were performed to evaluate the damage of target tumor cells at E/T ratio of 10:1. After 18 h incubation with B7-H3Bi-armed ATC or unarmed ATC, as shown in Figure 7B, the concentration of LDH with armed effectors was significantly greater than that with unarmed effectors in B7-H3-positive tumor cells (a). Moreover, a significant increase was observed in IFN-γ (b) and TNF-α (c) secretion by B7-H3Bi-armed ATC over their unarmed ATC counterpart when ATC was co-cultured with B7-H3-positive tumor cell respectively (*P* < 0.05). Although there was an increased secretion of IL-2 by B7-H3Bi-armed ATC compared with their unarmed ATC counterpart in B7-H3-positive tumor cells, it was not significantly (d). Meanwhile, as shown in Figure 7C, real-time photographs demonstrated that B7-H3Bi-armed ATC, but not unarmed ATC, aggregated with B7-H3-positive, but not B7-H3-negative primary tumor cells, clustering around the edge of targeting cell bulk which showed the specific lysis of B7-H3Bi-armed ATC.

## DISCUSSION

Although the first Bi-Ab was produced more than two decades ago, till recently Bi-Abs redirecting activated T cell to TAA has shown encouraging antitumor effects in both preclinical and clinical studies [6–9, 27, 28]. The major advantages of this approach include the large numbers of ATC can be produced ex vivo, and the mAbs specific for the surface TAA [4].

B7-H3 overexpression in human cancers appears to be a common phenomenon, therefore, B7-H3 may represent an attractive target for diagnostic and therapeutic manipulation. In this study, we tested whether B7-H3 is a useful target for the development of novel anti-CD3 x anti-B7-H3 therapeutics for cancers. It was found that B7-H3Bi-armed ATC secreted more IFN-γ, TNF-α and IL-2 than unarmed ATC, and had enhanced cytotoxic activity against a wide range of human cancer cells, including lung cancer, breast cancer, colorectal cancer, pancreatic cancer, cervix cancer, prostate cancer, and glioblastoma. Consistent with these in vitro results, our in vivo studies showed that infusion of B7-H3Bi-armed ATC remarkably inhibited tumor growth and prolonged survival time in both subcutaneous and lung metastatic xenograft mice.

The present study demonstrated that B7-H3Bi-armed ATC mediated higher level of specific cytotoxicity against B7-H3-positive tumor cells, compared with ATC alone, anti-B7-H3 mAb alone, or control unarmed ATC. These results indicated that T-cell cytotoxicity was dependent upon the engagement of B7-H3 TAA via B7-H3Bi-Ab bridge. Indeed, at the E/T ratio of 10:1, 5 ng Bi-Ab per 10^6^ ATC showed more than 60% cytotoxicity against Hela-luc cell after 18 h incubation, whereas anti-B7-H3 antibody alone had no inhibitory effect till the concentration of 5 μg/ml after 72 h incubation (Supplementary Data 2). In addition, our data supported that arming ATC with B7-H3Bi-Ab bypassed the requirement for major histocompatibility complex antigen recognition by T cell [29, 30]. Notably, human B7-H3Bi-armed ATC could not kill B7-H3-negative human tumor cell or mouse lung cancer cell, suggesting the specificity of B7-H3Bi-Ab.

Upon incubating B7-H3Bi-armed ATC with the tumor cells, we detected high levels of IFN-γ and TNF-α secreted by B7-H3Bi-armed ATC. These cytokines are known to be released by activated T cells and tumoricidal directly. It is noteworthy that in our in vitro cytotoxicity assays, B7-H3Bi-armed ATC mediated cell killing effectively without the addition of IL-2 to the cultures. The increased secretion of cytokines implicates that armed ATC infusion can recruit patient resident T cell and antigen presenting cell to process their own tumor lysates. This finding is in accordance with phase I clinical trials of Her2Bi-armed ATC in patients with stage IV breast cancer and hormone refractory prostate cancer; these patients showed elevated levels of cytokines in the serum, suggesting armed ATC administration stimulated the endogenous immunity to develop antitumor activity [29, 31, 32].

Our in vivo studies demonstrated that, compared with unarmed ATC control, B7-H3Bi-armed ATC significantly inhibited tumor growth in both loco-regional and systemic SCID xenograft models. Although B7-H3Bi-armed ATC could not eradicate the tumor completely, they prolonged the survival time of mice bearing established B7-H3-positive subcutaneous or lung metastatic tumors. Considering the hostile xenograft environment for human immune cells, infusions of armed ATC may be insufficient. Function persistence and survival of human ATC in xenograft model are more difficult than those in the immune system of the patients themselves. Lum LG group demonstrated that the cytotoxic activity of human T cells armed with Bi-Ab were durable with repeated tumor stimulation [33]. Treatment with B7-H3Bi-armed ATC, in combination with immune checkpoint blockade, may be more beneficial for the patients [2, 5, 34].

In conclusion, our in vitro and in vivo results suggest that B7-H3Bi-armed ATC may be a promising method for the treatment of B7-H3-positive cancer patients and produce clinically significant antitumor effects.

## MATERIALS AND METHODS

### Cell lines, primary culture and mice

A549-luc, BXPC-3-luc, HT-29-luc, Hela-luc, MDA-MB231-luc, NCIH460-luc, PC-3M-luc, and LL/2-luc-M38 cell lines were all purchased from Caliper Life Sciences. U87MG-luc cells were generated by our group previously [35]. The primary culture was derived from freshly isolated tumor cell from patients of two primary lung cancer or two primary breast cancer individually, in Beijing Shijitan Hospital of Capital Medical University. The consent was obtained from the patients before sample collection. The study complied with the Declaration of Helsinki and was approved by the Biomedical Research Ethics Committee of Beijing Shijitan Hospital of Capital Medical University. Briefly, tumors were placed into RPMI1640 medium with antibiotics and anti-fungal immediately following resection. Specimens were washed with cold PBS repeatedly to remove blood clots and tissue debris, and then minced into 0.5- to 1.0-mm pieces. The tissue pieces were digested with collagenase IV, Dnase I and hyaluronidase. Then cells were washed and passed through a 70 μm cell strainer to obtain single-cell population. All agents for cell culture were from Gibco Company (Gaithersburg, MD, USA). Beige-SCID Mice (8 to 10 weeks) were purchased from Vital River Laboratories (VRL, Beijing, China).

### Isolation of peripheral blood lymphocytes and preparation and cryopreservation of activated T lymphocytes (ATC)

Peripheral mononuclear blood cells (PBMCs) were isolated using Ficoll density gradient centrifugation from healthy donors supplied by the Beijing Blood Bank. PBMCs were cultured at 1×10^6^/ml in RPMI-1640 medium supplemented with 10% FBS and 5 μg/ml anti-CD3 mAb (eBioscience, San Diego, CA, USA) and 100 IU/ml recombinant human IL-2 at 1×106/ml. Fresh medium containing 100 IU/ml recombinant human IL-2 was added to cell culture as the method previously described [27, 28, 36]. On day 13, ATC expansion products of donors averaged 97.33 ± 0.86% CD3^+^ cells (32.53± 3.00% CD4^+^ and 64.87 ± 0.81% CD8^+^) were used immediately or cryopresered for further use.

### Synthesis of anti-CD3 ×anti-B7-H3 bispecific antibody (B7-H3Bi-Ab) and arming of activated T cell

Anti-B7-H3 mAb (R&D System, Minneapolis, MN, USA) was reacted with sulfo-SMCC and anti-CD3 (OKT3, eBioscience) was reacted with Traut's reagents as the method previously described [27, 28, 35]. Cryopreserved ATC was thawed, and usually armed with B7-H3Bi-Ab at a concentration of 50 ng/10^6^ cells at room temperature for 30 minutes followed by washing the cells to eliminate unbound antibodies. The combination of OKT3 (50 ng/10^6^cells) with anti-B7-H3 mAb (50 ng/10^6^cells) pre-incubated activated T cell was used as control unarmed ATC.

### Cytotoxicity assay

Target cells were seeded in triplicates in 96-well u bottom microplates at 1×10^4^/well before the addition of B7-H3Bi-armed, unarmed ATC or ATC at the indicated effector-to-target (E/T) ratios. Effector cells and tumor cells were allowed to interact at 37°C for 18 hours. Cytotoxicity was mostly measured with a luciferase quantitative assay [27, 28, 35, 37, 38] in which bioluminescence imaging signal in tumor cells expressed in photons per second was converted into living cell number, and the cytotoxicity assays were calculated at indicated E/T ratios. A final concentration of 0.15 mg/ml D-luciferin (SynchemChemie, Kassel, Germany) was added to each well. Sometimes, when the target cells were freshly isolated tumors from primary culture, cytotoxicity assays were performed by a lactate dehydrogenase activity assay kit (Sigma-Aldrich, St. Louis, MO, USA) according to the manufacturer's instructions, and real-time photograph was taken at x 200 magnification with an optical microscope (Olympus, Tokyo, Japan).

### Cytokine ELISA assay

Target cells were plated in 96-well u bottom microplates at a concentration of 1×10^4^/well at 37°C overnight. B7-H3Bi-armed, unarmed ATC or ATC was then added at an E/T ratio of 10:1 to target cells and incubated for 18 hours. The cell free supernatants were collected, and the cytokine production of IFN-γ, TNF-α and IL-2 was measured by using specific human ELISA kit (eBioscience) according to the manufacturer's instructions.

### Flow cytometric analysis

The anti-human B7-H3-PE mAb and mouse IgG1-PE isotpye antibody were purchased from R&D System. The anti-human CD3-FITC, anti-mouse IgG1-FITC, and anti-mouse IgG2a-FITC secondary antibodies were provieded by eBioscience. The cells were assayed with a Guava flow cytometrometer Easycyte (Guava, Hayward, CA, USA) and the data analysis was carried out with the FlowJo Software Version 7.6.1.

### In vivo tumor inhibition studies

In tumor growth delay studies, SCID-Beige mice were injected s.c. with Hela-luc or i.v. with A549-luc cells. Subsequently, B7-H3Bi-armed ATC or control unarmed ATC was administrated on the indicated day. In order to follow up tumor growth, in vivo bioluminescence imaging was operated and bioluminescent imaging was done using Xenogen IVIS-100 imaging system with living image software (Caliper Life Science). The signal intensity of tumor burdens was expressed as total photons/second/cm^2^ *(p/s/cm^2^/sr.)*. Animal experiment was in compliance with the Guide for the Care and Use of Laboratory Animals of the Ministry of Health and the protocol was approved by the Ethics Committee of Beijing Shijitan Hospital of Capital Medical University.

### Statistical analysis

All experiments were repeated at least twice and mostly three times. Data were analyzed using Graphpad Prism 5 software (Graphpad, La Jolla, CA), and presented as the means ± SDs. Unpaired student's *t*-test (two-tailed) was used for comparison of two groups where appropriate. Survival curves were plotted using the Kaplan-Meier method and compared using the log-rank test. *P* < 0.05 was considered as statistically significant.

## SUPPLEMENTARY DATA FIGURES


