# Supplementary material for: B7-H3 as a promising target for cytotoxicity T cell in human cancer therapy
Source: Oncotarget. 2016 Apr 18;7(20):29480–91. doi: 10.18632/oncotarget.8784 (PMC5045411; doi:10.18632/oncotarget.8784)
Supplement: Supplementary file 1 [file oncotarget-07-29480-s001.pdf]

# SUPPLEMENTARY DATA

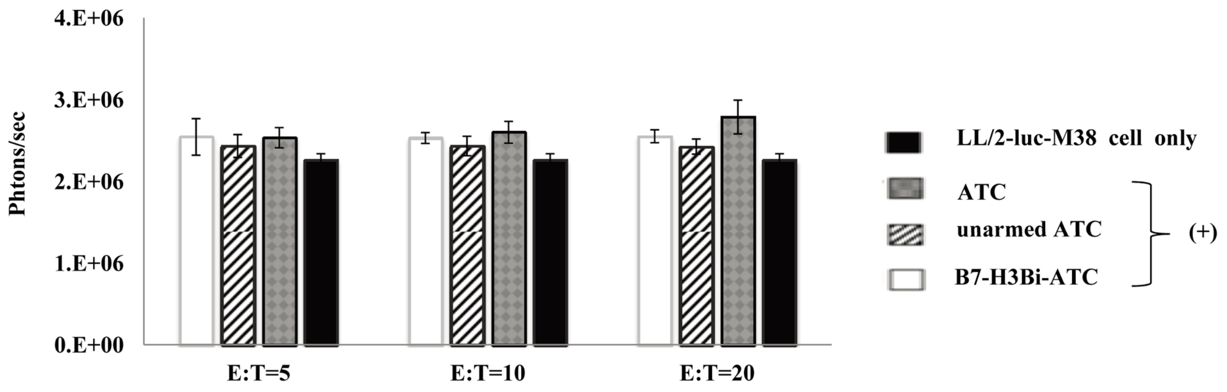


**Supplementary Data S1: The cytotoxicity effects of B7-H3Bi-armed ATC against mouse lung cancer cell.** Target mouse lung cancer cell LL/2-luc-M38 was incubated either with B7-H3Bi-armed ATC or unarmed ATC, or ATC for 18 hours, and luciferase quantitative assay was performed to determine cytotoxicity against target cell at different E/T ratio. The data are mean ± SD of triplicate determination. Shown is a representative experiment of at least three.

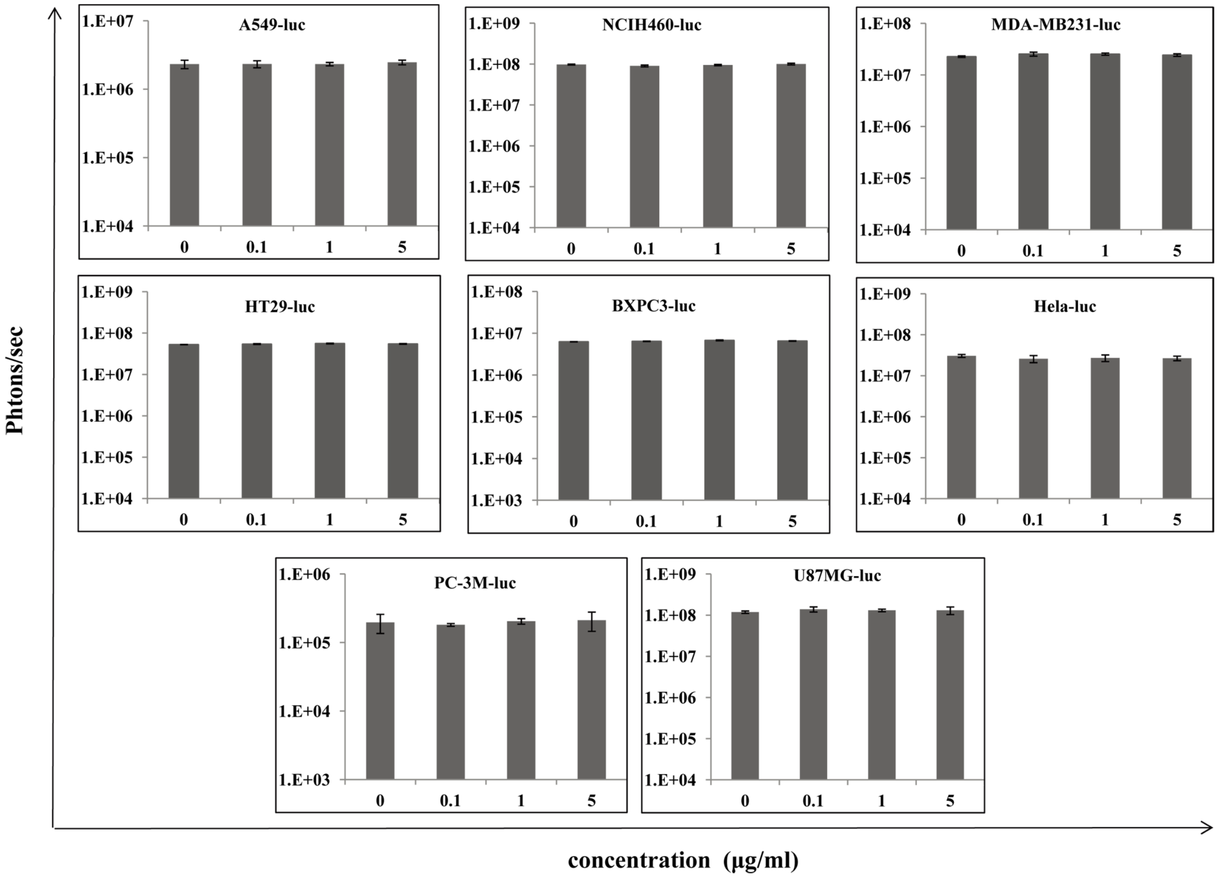


**Supplementary Data S2: The cytotoxicity effects of anti-human B7-H3 mAb on tumor cells.** Different human B7-H3-positive tumor cells were treated with or without anti-human B7-H3 mAb at indicated concentration for 72 h, and luciferase quantitative assay was performed to determine the cytotoxicity effects of anti-human B7-H3 mAb on tumor cells. The data are mean ± SD of triplicate determination. Shown is a representative experiment of at least three.
